# Bleeding Risk and Mortality of Edoxaban: A Pooled Meta-Analysis of Randomized Controlled Trials

**DOI:** 10.1371/journal.pone.0095354

**Published:** 2014-04-15

**Authors:** Shuang Li, Baoxin Liu, Dachun Xu, Yawei Xu

**Affiliations:** Department of Cardiology, Shanghai Tenth People's Hospital, Tongji University School of Medicine, Shanghai, China; Universidad Peruana de Ciencias Aplicadas (UPC), Peru

## Abstract

**Objective(s):**

Edoxaban, a factor Xa inhibitor, is a new oral anticoagulant that has been developed as an alternative to vitamin K antagonists. However, its safety remains unexplored.

**Methods:**

Medline, Embase and Web of Science were searched to March 8, 2014 for prospective, randomized controlled trials (RCTs) that assessed the safety profile of edoxaban with warfarin. Safety outcomes examined included bleeding risk and mortality.

**Results:**

Five trials including 31,262 patients that met the inclusion criteria were pooled. Overall, edoxaban was associated with a significant decrease in major or clinically relevant nonmajor bleeding events [risk ratio (RR) 0.78, 95% confidence interval (CI) 0.74 to 0.82, p<0.001] and any bleeding events [RR 0.82, 95% CI 0.79 to 0.85, p<0.001]. Edoxaban also showed superiority to warfarin both in all-cause mortality [RR 0.92, 95% CI0.85 to0.99, p = 0.02] and cardiovascular mortality [RR 0.87, 95% CI0.79 to 0.96, p = 0.004]. Subgroup analyses indicated that RRs of edoxaban 30, 60 or 120 mg/d were 0.67 (p<0.001), 0.87 (p<0.001) and 3.3 (p = 0.004) respectively in major or clinically relevant nonmajor bleeding; 0.71 (p<0.001), 0.89 (p<0.001) and 2.29 (p = 0.002) respectively in any bleeding; as well as 0.86 (p = 0.01), 0.87 (p = 0.01) and 0.28 (p = 0.41) respectively in cardiovascular death… Meanwhile, paramount to note that pooled results other than the largest trial showed edoxaban was still associated with a decrease in the rate of major or clinically relevant nonmajor bleeding event (p = 0.02) and any bleeding (p = 0.002), but neither in all-cause death (p = 0.66) nor cardiovascular death (p = 0.70).

**Conclusions:**

Edoxaban, a novel orally available direct factor Xa inhibitor, seems to have a favorable safety profiles with respect to bleeding risk and non-inferior in mortality when compared to warfarin. Further prospective RCTs are urgently needed to confirm the results of this meta-analysis.

## Introduction

For many decades, vitamin K antagonists (VKAs) were the only available therapy for long-term anticoagulation.[1], [2] However, VKAs exhibit a considerable variability in dose response among patients, participate in multiple food and drug interactions, and have a narrow therapeutic window.[3], [4] These limitations has prompted the development of a series of new oral anticoagulants (OACs) as alternatives to VKAs, including direct thrombin inhibitors such as dabigatran as well as direct factor Xa inhibitors including rivaroxaban, apixaban, and edoxaban. These new OACs appear to offer practical advantages over VKAs, with fewer food and drug interactions, a fixed daily or weekly dose, and no need for monitoring of the anticoagulant effect.[5] Several large randomized clinical trials (RCTs) have already been compared these new OACs with VKAs and two trials were cited by the European Society of Cardiology (ESC) to recommend a recently updated guideline for dabigatran and rivaroxaban as preferable to VKA for preventing stroke and other thromboembolic events in the vast majority of people with atrial fibrillation (AF) [6].

Edoxaban is a latest factor Xa inhibitor with several studies investigating the efficacy and safety for different indications. However, the risk for bleeding and mortality associated with this drug remains unexplored comprehensively. We therefore performed a systematic meta-analysis to compare the safety of rivaroxaban with standard VKAs therapy (warfarin), particularly focusing on bleeding and mortality.

## Materials and Methods

### Search Criteria

We performed a computerized search to identify relevant RCTs using Medline (via PubMed, from inception to March 8, 2014), Embase (via OVID, from 1966 to 2014), and Web of Science (including databases of SCI-EXPANDED, SSCI, A&HCI, CPCI-S, CCR-EXPANDED, IC, from 1984 to 2014) for comparing the safety of edoxaban with warfarin. We used the following keywords: “new oral anticoagulants”, “edoxaban”, “factor Xa inhibitor”, and “Warfarin” No language restrictions. Publication type was limited to be RCT. We also attempted to contact authors of included study, and even asked a product manager of Daiichi Sankyo Pharma Development, the manufacturer of edoxaban for any unpublished data.

### Study selection

Studies were eligible to be included in our meta-analysis if they (1) were prospectively randomized patients to receive either edoxaban or warfarin (2) had treatment duration for at least 3 months (3) had certain safety outcomes the events of bleeding risk or mortality. No restrictions were placed on population size or languages. We excluded studies that were retrospective or nonrandomized or those in which patients were not randomized to receive the edoxaban used. Letters to the editor, editorials, reviews, and abstracts from conference proceedings were also excluded from our study. All studies were reviewed independently by Dr. Yawei Xu and Dr. Dachun Xu, who have more than 30 and 20 years respectively of experience as electrophysiological cardiologists to determine whether they match the eligibility for inclusion. A kappa value was calculated to assess the degree of agreement.

### Data extraction

Data were independently extracted by another two reviewers (Shuang Li, Baoxin Liu) and disagreements were resolved by consensus. Attempts were made to retrieve the data directly from the published papers or sent mails to authors for acquiring data not published. Demographic and clinical characteristics of each trial were recorded, including age, gender, numbers of subjects, information about hypertension, diabetes, congestive heart failure, previous warfarin use, prior stroke, each event of bleeding and mortality from included trials.

### Risk of bias in included studies

We used the Cochrane Collaboration's recommended tool for assessing the risk of bias in included studies [7]. Trials' quality was assessed by evaluating every element of study design: blinding description, randomization process, inclusion and exclusion criteria, concealed allocation, intention-to-treat analysis, and assessment of withdrawals and dropouts. Risk for bias was assessed in duplicate, with disagreements resolved by consensus.

### Assessment of Heterogeneity

We tested heterogeneity between trial results with the Cochrane Chi-square test and I^2^ statistics (percentage of total variation across studies due to heterogeneity). A I^2^ of 0–25% indicates no observed heterogeneity, and larger values show increasing heterogeneity, with 25–50% defined as low, 50–75% as moderate, and above 75% as high heterogeneity, respectively [8].

### Data Synthesis and Analysis

All analyses were performed with review manager software (RevMan Analyses Version 5.2.4 Copenhagen; The Nordic Cochrane Center, The Cochrane Collaboration, 2013). The primary safety end-points of our meta-analysis were bleeding events (major or clinically relevant non-major bleeding event, any bleeding events) and mortality (all-cause death, cardiovascular death for patients received edoxaban or warfarin. Meanwhile, we also reported major bleeding event, clinically relevant non-major bleeding and minor bleeding.

Subgroup analyses of different fixed doses of edoxaban were performed. We calculated a weighted estimate of the typical treatment effect across trials using risk ratio (RR) by means of a fixed-effect model. However, in the study with moderate to high heterogeneity (I^2^>50%), a random-effect model was performed. RRs and their two-sided 95% confidence intervals (CI) were reported. A 95% CI not including 1 and p<0.05 were considered statistically significant.

## Results

### 1. Literature searching

**Figure 1:**
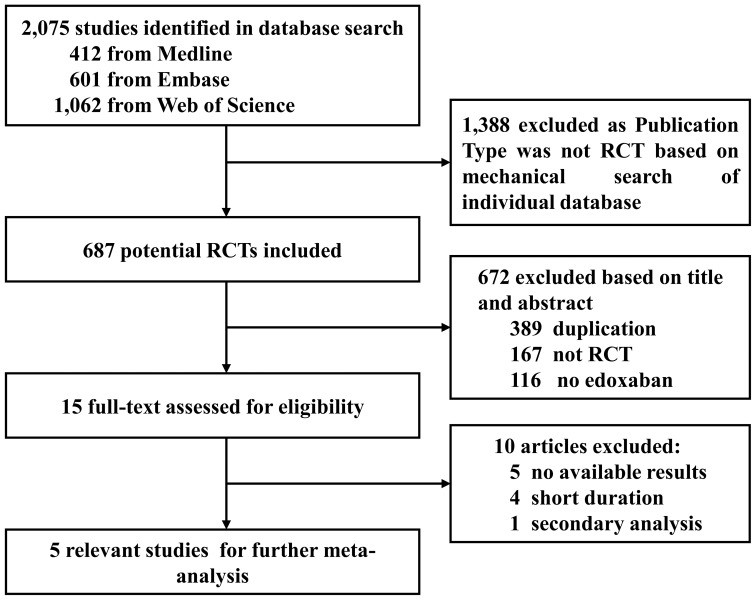
Flow Diagram of Selection Strategy. Flow diagram depicting the selection strategy for trials used in this meta-analysis. Please note that when we meant the phrase of ‘no available data’, we meant that there was no associated result that matched the end-point outcomes of our meta-analysis. **RCT** denotes randomized clinical trial, **SCIE** Science Citation Index Expanded databases.

As shown in Figure 1, three databases were searched until March 8, 2014 (Table S1–3). A total of 2,075 articles were reviewed, of which1, 388 articles were initially rejected because they were not RCTs based on mechanical search in individual database. Then 672 potential ones were excluded based on title and abstract. Of the rest of 15 remaining studies with full-text assessment, 10 had no available data or short duration <3 months or were secondary analysis (Table S4), that might be no association with potential bias. Finally, five RCTs [9], [10], [11], [12], [13] met our inclusion criteria and were included in our study. No additional data was found either from authors' responses or the internal database of Daiichi Sankyo Pharma Development, the manufacturer of edoxaban. All included processes were performed independently by Dr. Yawei Xu and Dr. Dachun Xu. The kappa value was 0.82, reflecting excellent agreement.

### 2. The methodological quality of the included trials

We assessed quality of the included trials using the Cochrane Collaboration's recommended tool for assessing the risk of bias in included studies[7]. Overall, all 5 trials were designed to be randomized and double-blind with a relatively low risk for bias (Table 1).

**Table 1:** Risk of bias in included studies.

Study	Type of Blinding	Method of Blinding Described and Appropriated	Randomization Process Described and Adequate	Adequate Concealed Allocation	Description of Withdrawals and Dropouts	Intention-to-Treat Analysis Performed	Important Baseline Differences Present	Inclusion and Exclusion Criteria Specified
ENGAGE AF-TIMI 48 [9]	Double	Yes	Yes	Yes	Yes	Yes	Yes	Yes
Hokusai-VTE [10]	Double	Yes	Yes	Yes	Yes	Yes	Yes	Yes
Yamashita, 2012 [11]	Double	Yes	NR	Yes	Yes	NR	Yes	Yes
Chung, 2011 [12]	Double	Yes	Yes	Yes	Yes	Yes	Yes	Yes
Weitz, 2010 [13]	Double	Yes	Yes	Yes	Yes	Yes	NR	NR

Risk of bias in included studies were evaluated every element of study design: blinding description, randomization process, inclusion and exclusion criteria, concealed allocation, intention-to-treat analysis, and assessment of withdrawals and dropouts. **NR** =  not reported.

### 3. Characteristics of patients and trials

A total of 31,262 subjects were included. Among the included studies, sample sizes ranged from 235[12] to 21,105[9]. Patients were predominantly men and received treatment for nonvalvular atrial fibrillation (NVAF, n = 23,022[9], [11], [12], [13]), deep vein thrombosis (DVT, n = 4,921[10]), or pulmonary embolism (PE, n = 3,319[10]). The median treatment duration ranged from 12 weeks (3 months) [11], [12], [13] to 907[9] days and follow-up ranged from 2 months [11] to 1022 days[9]. Efficacy endpoints differed among those studies; however, safety outcomes (i.e., bleeding or mortality) were included. Safety analyses included all patients who received more than 30 mg/d dose of edoxaban or open-label adjusted dose of warfarin, maintaining international normalized ratio (INR) 2–3. (Table 2)

**Table 2:** Patient- and study-level characteristics of randomized controlled trials comparing edoxaban to warfarin.

Characteristics	ENGAGE AF-TIMI 48		Hokusai-VTE		Yamashita, 2012		Chung, 2011		Weitz, 2010	
	Edoxaban	Warfarin	Edoxaban	Warfarin	Edoxaban	Warfarin	Edoxaban	Warfarin	Edoxaban	Warfarin
**Year**	**2013**		**2013**		**2012**		**2011**		**2010**	
**Country**	**1393 centers in 46 countries**		**439 centers in 37 countries**		**61 centers in Japan**		**4 Asian countries**		**91 centers in 12 countries**	
**Period**	**2008.11**–**2010.11**		**2010.1**–**2012.12**		**2007.4**–**2008.7**		**2007.10**–**2008.10**		**2007.7**–**2010.10**	
**Study Design**	**RCT, phase III**		**RCT, phase III**		**RCT, phase II**		**RCT, phase II**		**RCT, phase II**	
**Population**	**NVAF**		**DVT±PE**		**NVAF**		**NVAF**		**NVAF**	
**Subjects, n**	**21105**		**8240**		**536**		**235**		**1146**	
**Dose**	**30/60 mg/d, QD**	**adjusted (INR 2**–**3)**	**30/60 mg/d, QD**	**adjusted (INR 2**–**3)**	**30,45,60 mg/d, QD**	**adjusted (INR 2**–**3 for age <70;1.6**–**2.6 for age ≥70)**	**30,60 mg/d, QD**	**adjusted (INR 2**–**3)**	**30,60 mg/d, QD or BID**	**adjusted (INR 2**–**3)**
**Age (year)** *	**72(64**–**78)**	**72(64**–**78)**	**55.7±16.3**	**55.9±16.2**	**69.1**	**68.8**	**64.5±9.5**	**61.5±8.5**	**65±8.7**	**66±8.75**
**Male/Female, %**	**61.6/38.4**	**62.5/37.5**	**57.3/42.7**	**57.2/42.8**	**82.3/17.7**	**83/17**	**66.7/33.3**	**62.7/37.3**	**62.6/37.4**	**60.4/39.6**
**Previous warfarin use, %**	**59**	**58.8**	**NR**	**NR**	**84.3**	**86**	**50.3**	**54.7**	**64.3**	**64.8**
**DM, %**	**36.3**	**35.8**	**NR**	**NR**	**20.2**	**31**	**32.7**	**22.7**	**NR**	**NR**
**HTN, %**	**93.6**	**63.9**	**NR**	**NR**	**73.5**	**71.3**	**72.3**	**69.3**	**NR**	**NR**
**Prior stroke or TIA, %**	**28.3**	**28.3**	**NR**	**NR**	**27**	**30.2**	**25.2**	**22.7**	**NR**	**NR**
**Congestive HF, %**	**57.4**	**57.5**	**NR**	**NR**	**25.3**	**33.3**	**27**	**32**	**NR**	**NR**
**Treatment duration**	**907 days (Medium)**		**3**–**12 months**		**12 weeks**		**3 months**		**3 months**	
**Follow-up**	**1022 days (Medium)**		**12 months**		**8 weeks**		**3 months**		**3 months**	

*measure as mean ±SD or median (interquartile range).

NVAF denotes nonvalvular atrial fibrillation; DVT deep vein thrombosis; PE pulmonary embolism; INR international normalized ratio ; QD que die; BID bis in die; NA not applicable; VKA vitamin K antagonist; TIA transient ischemic attack; HF heart failure; DM diabetes mellitus; HTN hypertension.

### 4. Outcome Measures Reporting

#### 4.1 Definitions of Bleeding

The trials included in our study reported several bleeding and mortality outcomes (Table 3). Across all included studies, bleeding events were reported including major or clinically relevant nonmajor bleeding event, major bleeding (any, fatal, gastrointestinal and intracranial), clinically relevant nonmajor bleeding, minor bleeding, fatal bleeding, any bleeding et al. All trials stated the declaration that all suspected bleeding events were assessed by an independent blinded adjudication committee.

**Table 3:** Study outcomes as reported in randomized controlled trials comparing edoxaban to warfarin.

Outcome*	ENGAGE AF-TIMI 48^7^		Hokusai-VTE^8^		Yamashita, 2012^9^		Chung, 2011^10^		Weitz, 2010^11^	
	Edoxaban (n = 14014)	Warfarin (n = 7012)	Edoxaban (n = 4118)	Warfarin (n = 4112)	Edoxaban (n = 394)	Warfarin (n = 125)	Edoxaban (n = 159)	Warfarin (n = 75)	Edoxaban (n = 893)	Warfarin (n = 250)
**Major or clinically relevant nonmajor bleeding event, n**	**2689**	**1761**	**349**	**423**	**16**	**4**	**6**	**5**	**54**	**8**
**Major bleeding, n**										
**Any**	**672**	**524**	**56**	**66**	**5**	**0**	**0**	**2**	**12**	**1**
**Fatal**	**52**	**59**	**2**	**10**	**NR**	**NR**	**NR**	**NR**	**NR**	**NR**
**Gastrointestinal.**	**361**	**190**	**1**	**2**	**NR**	**NR**	**NR**	**NR**	**NR**	**NR**
**Intracranial-Fatal**	**36**	**42**	**NR**	**NR**	**NR**	**NR**	**NR**	**NR**	**NR**	**NR**
**Intracranial-Any**	**102**	**132**	**0**	**6**	**NR**	**NR**	**NR**	**NR**	**NR**	**NR**
**Clinically relevant nonmajor bleeding, n**	**2183**	**1396**	**298**	**368**	**NR**	**NR**	**6**	**3**	**42**	**7**
**Minor Bleeding, n**	**1137**	**714**	**NR**	**NR**	**76**	**21**	**48**	**17**	**NR**	**NR**
**Any bleeding, n**	**3564**	**2114**	**895**	**1056**	**90**	**25**	**35**	**22**	**94**	**20**
**Any-cause death, n**	**1612**	**839**	**132**	**126**	**NR**	**NR**	**NR**	**NR**	**NR**	**NR**
**Cardiovascular mortality, n**	**1057**	**611**	**15**	**12**	**NR**	**NR**	**NR**	**NR**	**6**	**2**

*Event rates were based on the intention-to-treat population unless otherwise specified.

Definitions of bleeding event (major bleeding, clinically relevant nonmajor bleeding event and minor bleeding) among the included trials were similar. Major bleeding was defined as bleeding that was fatal or in a critical site (intracranial, intraocular, intraspinal, retroperitoneal, intra-articular, pericardial, or intramuscular with compartment syndrome) or overt and associated with a decline in haemoglobin of ≥2 g/dl or requiring transfusion of ≥2 units of blood [9], [10], [11], [13], which consistent to the definition by the International Society on Thrombosis and Haemostasis [14] or plus transfusion≥800 ml of packed red blood cells or whole blood [12]. Clinically relevant nonmajor bleeding was defined as overt bleeding that did not meet the criteria for major bleeding but was associated with the need for medical intervention [9], [10], or did not meet the criteria for major bleeding but consisting of hematoma≥5 cm in diameter/≥25 cm2; epistaxis or gingival bleeding ≥5 min in the absence of external factors[11], [12], [13]. Minor bleeding was defined as any bleeding that did not meet the criteria for a major or clinically relevant nonmajor bleeding event [9], [13] and included macroscopic haematuria; occult haematuria≥2+; occult haematuria with microscopic (RBC)≥10/high power field; ecchymosis, epistaxis and gingival bleeding occurring without any external stimuli [11], [12]. Fatal bleeding was not separately defined [9], [10].

#### 4.2 The primary outcomes

**Figure 2:**
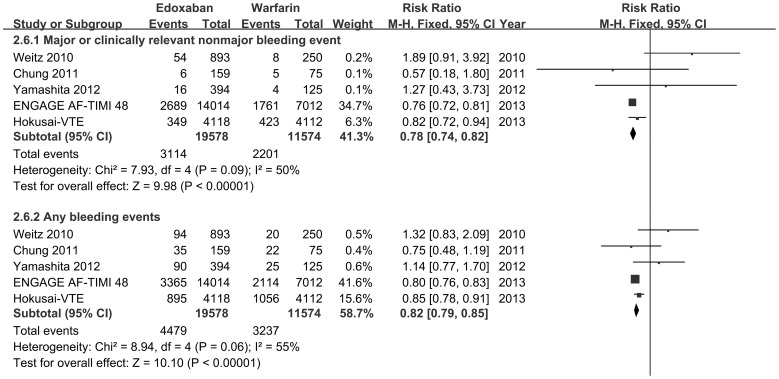
Forest Plot of risk ratios of bleeding events for comparison edoxaban with warfarin. A series of forest plots of risk ratios (RRs) of bleeding events for comparison of given edoxaban or warfarin according to every trial were pooled. All five trials (n = 31,262) reported events of major or clinically relevant nonmajor bleeding event and any bleeding. CI confidence interval.

All 5 trials (19,578 received edoxaban and 11,574 received warfarin) reported events of major or clinically relevant nonmajor bleeding event, and any bleeding. When data were pooled across the included studies, we found that edoxaban was associated with a decrease in major or clinically relevant nonmajor bleeding [RR 0.78, 95% CI0.74 to 0.82, p<0.001] and any bleeding events [RR 0.82, 95% CI 0.79 to 0.85, p<0.001]. (Figure 2)

**Figure 3:**
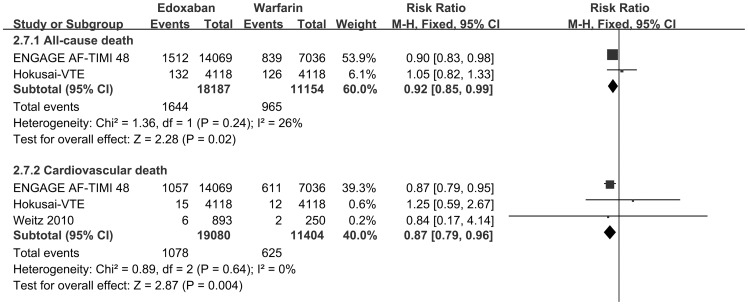
Forest plots of studies for mortality for comparison edoxaban with warfarin. Forest plots of studies for mortality (from all causes or cardiovascular disease) for comparison edoxaban with warfarin. Two trials (n = 29,256) reported available data. CI confidence interval.

Across all 5 studies, 2 trials[9], [10] (18,132 receives edoxaban and 11,124 received warfarin) reported available events of all-cause death, and 3 trials[9], [10], [13] (19,025 receives edoxaban and 11,374 received warfarin) reported events of death as for cardiovascular disease (CVD). Edoxaban also showed superiority to warfarin in reduction rates of both all-cause death [RR 0.92, 95% CI 0.85 to 0.99, p = 0.02] and cardiovascular death [RR 0.87, 95% CI 0.79 to 0.96, p = 0.004]. (Figure 3)

Additionally, we also reported that edoxaban was associated with a decrease in any major bleeding [RR 67, 95% CI 0.60 to 0.74, p<0.001], clinically relevant nonmajor bleeding [RR 0.79, 95% CI 0.75 to 0.84, p<0.001], minor bleeding [RR 1.04, 95% CI 0.95 to 1.14, p = 0.35] and fatal bleeding [RR 0.42, 95% CI 0.29 to 0.60, p<0.001]. (Figure S1)

#### 4.3 The Study of ENGAGE AF-TIMI 48

A pooled analysis of the other RCTs was also performed, other than ENGAGE AF-TIMI 48 (Figure S2 and S3), to compare with the total result. For bleeding risks, pooling results of other trials indicated consistence with the total ones. Edoxaban was still associated with a decrease in major or clinically relevant nonmajor bleeding event [RR 0.80, 95% CI 0.75 to 0.98, p = 0.02] and any bleeding [RR 0.87, 95% CI 0.80 to 0.93, P = 0.002] (Figure S2). For mortality, edoxaban showed no superiority to warfarin in reduction rate of either all-cause death [RR 1.17, 95% CI 0.59 to 2.31, p = 0.66] or cardiovascular death [RR 1.05, 95% CI 0.82 to 1.33, p = 0.66]. (Figure S3).

#### 4.4 Subgroup meta-analyses

**Figure 4:**
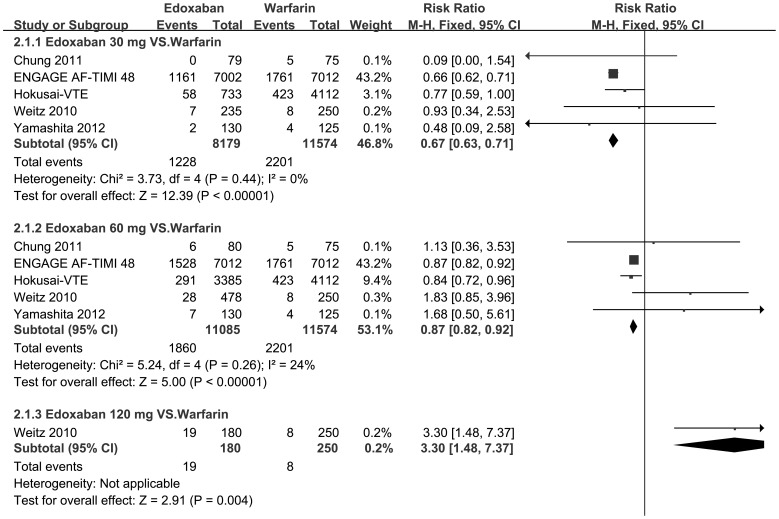
A series of forest plots of risk ratios of major or clinically relevant nonmajor bleeding event for comparison each fixed dose of edoxaban with warfarin. A series of forest plots of risk ratios (RRs) of major or clinically relevant nonmajor bleeding events for comparison each fixed dose of edoxaban (30, 60 or 120 mg per day) with warfarin if data were available. CI confidence interval.

**Figure 5:**
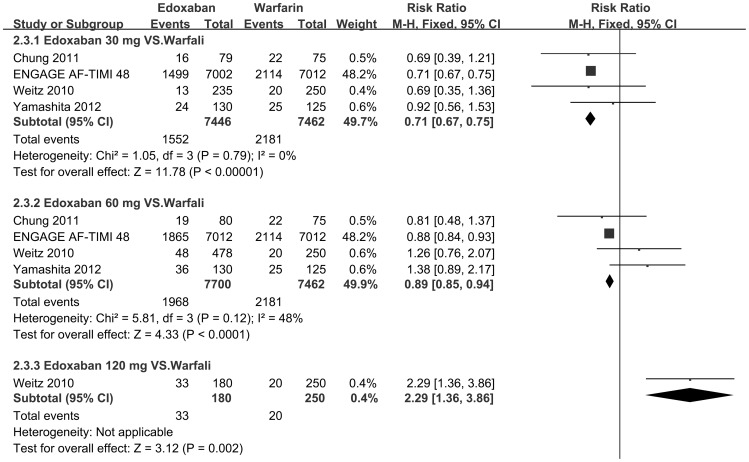
A series of forest plots of risk ratios of any bleeding event for comparison each fixed dose of edoxaban with warfarin. A series of forest plots of risk ratios (RRs) of any bleeding events for comparison each fixed dose of edoxaban (30, 60 or 120 mg/d) with warfarin if data were available. CI confidence interval.

**Figure 6:**
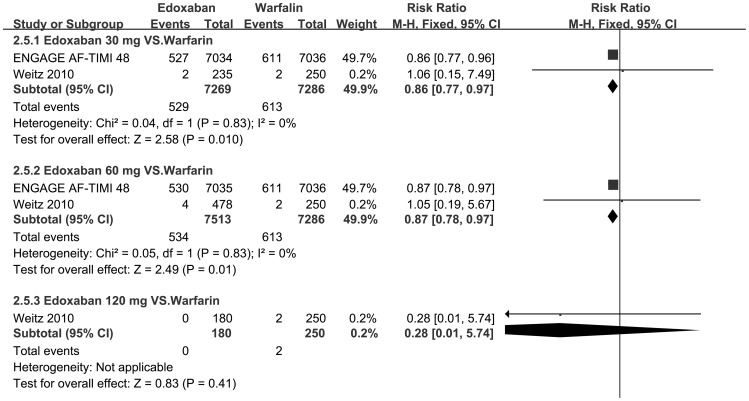
Forest plots of risk ratios for events of cardiovascular death for comparison each fixed dose of edoxaban with warfarin. Forest plots of risk ratios (RRs) for events of cardiovascular death for comparison each fixed dose of edoxaban with warfarin. CI confidence interval.

Furthermore, a series of subgroup meta-analyses of different fixed doses (30, 60 or 120 mg/d) of edoxaban in comparison to warfarin were conducted (Table 4, Figure 4–6). As for Weitz 2010[13], we defined the subgroup of “edoxaban 60 mg/d” as the combination of “edoxaban 30 mg bid” and “60 mg qd” in the original protocol for medication.

**Table 4:** Subgroup analyses of safety outcomes based on different fixed doses of edoxaban.

Edoxaban*	Warfarin	Trials	Edoxaban		Warfarin		RR	95% CI	P value	I^2^ (%)
			Event,n	Total,n	Event,n	Total,n				
**1. Major or clinically relevant nonmajor bleeding event**										
**30**	**Warfarin**	**Chung 2011**	**0**	**79**	**5**	**75**	**0.67**	**0.63**–**0.71**	**<0.001**	**0**
		**ENGAGE AF-TIMI 48**	**1161**	**7002**	**1761**	**7012**				
		**Hokusai-VTE**	**58**	**733**	**423**	**4112**				
		**Weitz 2010**	**7**	**235**	**8**	**250**				
		**Yamashita 2012**	**2**	**130**	**4**	**125**				
**60**	**Warfarin**	**Chung 2011**	**6**	**80**	**5**	**75**	**0.87**	**0.82**–**0.92**	**<0.001**	**24**
		**ENGAGE AF-TIMI 48**	**1528**	**7012**	**1761**	**7012**				
		**Hokusai-VTE**	**291**	**3385**	**423**	**4112**				
		**Weitz 2010**	**28**	**478**	**8**	**250**				
		**Yamashita 2012**	**7**	**130**	**4**	**125**				
**120**	**Warfarin**	**Weitz, 2010**	**19**	**180**	**8**	**250**	**3.3**	**1.48**–**7.37**	**0.004**	**NA**
**2. Any bleeding**										
**30**	**Warfarin**	**Chung 2011**	**16**	**79**	**22**	**75**	**0.71**	**0.67**–**0.75**	**<0.001**	**0**
		**ENGAGE AF-TIMI 48**	**1499**	**7002**	**2114**	**7012**				
		**Weitz 2010**	**13**	**235**	**20**	**250**				
		**Yamashita 2012**	**24**	**130**	**25**	**125**				
**60**	**Warfarin**	**Chung 2011**	**19**	**80**	**22**	**75**	**0.89**	**0.85**–**0.94**	**<0.001**	**48**
		**ENGAGE AF-TIMI 48**	**1865**	**7012**	**2114**	**7012**				
		**Weitz 2010**	**48**	**478**	**20**	**250**				
		**Yamashita 2012**	**36**	**130**	**25**	**125**				
**120**	**Warfarin**	**Weitz 2010**	**33**	**180**	**20**	**250**	**2.29**	**1.36**–**3.86**	**0.002**	**NA**
**3. Cardiovascular death**										
**30**	**Warfarin**	**ENGAGE AF-TIMI 48**	**527**	**7034**	**611**	**7036**	**0.86**	**0.77**–**0.97**	**0.01**	**0**
		**Weitz 2010**	**2**	**235**	**2**	**250**				
**60**	**Warfarin**	**ENGAGE AF-TIMI 48**	**530**	**7035**	**611**	**7036**	**0.87**	**0.78**–**0.97**	**0.01**	**0**
		**Weitz 2010**	**4**	**478**	**2**	**250**				
**120**	**Warfarin**	**Weitz 2010**	**0**	**180**	**2**	**250**	**0.28**	**0.01**–**5.74**	**0.41**	**NA**

*Dose measured as mg per day.

NA not application; RR risk ratio; CI confidence interval.

Generally, relatively lower dose (30 or 60 mg/d) was associated with a decrease both in bleeding risk (Figure 4–5) and cardiovascular mortality (Figure 6) in comparison to warfarin. The RRs of bleeding risk that received edoxaban 30, 60 and 120 mg/d were 0.67 [95% CI 0.63–0.71, p<0.001], 0.87 [95% CI 0.82–0.92, p<0.001] and 3.3 [95% CI 1.48–7.37, p = 0.004] respectively in major or clinically relevant nonmajor bleeding (Figure 4); 0.71 [95% CI 0.67–0.75, p<0.001], 0.89 [95% CI 0.85–0.94, p<0.001] and 2.29 [95% CI 1.36–3.86, p = 0.002] respectively in any bleeding events (Figure 5) to that of warfarin. Meanwhile, every fixed dose was non-inferior to warfarin on reduction rate of cardiovascular mortality (Figure 6). Given 30 mg and 60 mg showed superiority to warfarin (RR 0.86 [95% CI 0.77–0.97, p = 0.01] and 0.87 95% CI 0.78–0.97, p = 0.01] respectively, Figure 6) but no significant difference between each other (p = 0.94). (Table 4)

## Discussion

### What is edoxaban?

Our systematical meta-analysis was designed to compare the safety of edoxaban with that warfarin. Edoxaban is a novel, orally available, highly specific, reversible and direct factor Xa inhibitor. It has a linear and predictable pharmacokinetic profile and 62% oral bioavailability[9], [15]. It achieves maximum concentration within 1 to 2 hours, and 50% is excreted by the kidney [5], [16]. Like other factor Xa inhibitors, edoxaban has a series of favorable profiles, including fewer food and drug interactions, a fixed daily dose, and no need for monitoring of the anticoagulant effect [5], which appears to offer practical advantages over vitamin K antagonists (VKAs).

### Bleeding Risk

Prior RCTs have been performed to assess bleeding risk of which 4 RCTs [9], [11], [12], [13] included patients with nonvalvular atrial fibrillation and 1trial [10] with acute venous thromboembolism. Across all studies, 2 trials [11], [13] reported given 30 and 60 mg edoxaban were noninferior to warfarin on safety profiles in patients with nonvalvular atrial fibrillation, while 3 trials [9], [10], [12] found was associated with a significantly lower rate of bleeding. Yamashita [11] also found edoxaban 30, 45, and 60 mg/day was associated with a numerical increase in all bleeding across the dose range but not insignificantly. Hokusai-VTE [10] found similar outcomes of mortality between edoxaban and warfarin but ENGAGE AF-TIMI 48[9] pointed that edoxaban was associated with significantly lower rate of death from cardiovascular causes.

We pooled data from trials and found that (1) in comparison to traditional anticoagulation therapy with warfarin, edoxaban, a new factor Xa inhibitor, has a favorable safety profiles with respect to bleeding risk (major or clinically relevant non-major bleeding event, any bleeding event); (2) For incidence of bleeding event, it seems dose-response effect that lower dose is associated with less bleeding event significantly.

However, in spite of numerous benefits, there are concerns regarding the potential risk for bleeding with edoxaban in practice. Like other factor Xa inhibitors, rivaroxaban and apixaban, there are no standard antidotes for the reversal of edoxaban in general [17]. Some studies indicated that the availability of a reliable factor Xa assay [18] and specific reversal strategies [19] in urgent clinical situations could potentially improve the safety profile of edoxaban, but no particular strategy is well accepted in practice at this time [9]. Also, similar to edoxaban, other new OACs (i.e. dabigatran, rivaroxaban, apixaban) can also be given in fixed doses without routine laboratory monitoring and fewer drug–drug and food–drug interactions than warfarin.

### Mortality profiles

Dentali [20] confirmed there were small differences among these new OACs with respect to the prevention of ischemic stroke, myocardial infarction, bleeding, or death. Some prior meta-analyses of efficacy and safety of new OACs versus warfarin have performed. Dentali [21] retrieved 12 studies (3 with dabigatran, 4 with rivaroxaban, 2 with apixaban, and 3 with edoxaban) and reported new OACs significantly reduced total mortality, cardiovascular mortality, intracranial hemorrhage but not major bleeding. Harenberg [22] made a network meta-analysis of dabigatran, rivaroxaban and apixaban from 3 trials and showed there was no difference in all-cause mortality. Miller [23] pooled 3 RCTs given dabigatran, rivaroxaban and apixaban respectively in patients of atrial fibrillation and found that new OACs were more efficacious than warfarin for the prevention of stroke and systemic embolism in patients with AF and with a decreased risk for intracranial bleeding. However, direct studies are still needed in comparison edoxaban to other new OACs to explore whether these are real differences in clinical efficacy and safety.

We found that in comparison to warfarin, edoxaban has a favorable safety profiles with respect to mortality (both all-cause death and cardiovascular death, Figure 3). Each fixed dose, even the highest dose (120 mg/d), was non-inferior to warfarin on reduction cardiovascular mortality (Figure 6). Moreover, given 30 mg and 60 mg showed superiority to warfarin (p<0.001 both, Figure 6) but no significant difference between each other (p = 0.94).

### The Trial of ENGAGE AF-TIMI 48

ENGAGE AF-TIMI 48[9], as a large trial, accounted for about 67.5% of participants in the meta-analysis, and therefore its results drove much of the findings. Also, this study shows very promising results, those were almost consistent with the results of our meta-analyses. Thus, it was suspicious to wonder if it was valid of simply summing up the largest trial and smaller ones. For these considerations, a pooled sub-analysis of the other 4 trials, expect ENGAGE AF-TIMI 48[9], was conducted to compare with the total pooling. The results indicated the decrease rate of bleeding risk for edoxaban did not affect by the largest trial, while whether edoxaban could reduce mortality was largely affected. Thus, we summarized a relatively conservative conclusion that when compared with warfarin, edoxaban seems to be superior to reduce the rate of bleeding events, but non-inferior to reduce mortality, based on current evidence from RCTs.

### Study strengths and limitations

The strengths of this meta-analysis are the systematic electronic search, the search criteria without language limitation and use of two review authors independently to examine and select studies.

Our meta-analysis is subject to the limitations inherent to all meta-analysis. The major limitation of our study is that the results are based on the comprehensive data of trials with heterogeneous RCTs, including patients with atrial fibrillation [9], [11], [12], [13], deep vein thrombosis[10], and pulmonary embolism [10]. They also differed on population sizes, different protocols of medication, efficacy outcomes, treatment duration and follow-up. We have attempted to account for these differences by conducting subgroup analyses if data were available. However, some limitations still existed and cause potential bias.

Firstly, ENGAGE AF-TIMI 48, as a large trial, accounted for around 67.5% of participants in the meta-analysis, and therefore its results drove much of the findings. Secondary, we attempted to search any unpublished data through mails to authors of each included study and the manufacturer, however, found no additional data[24]. Beside, FDA was not requested for additional data. All five RCTs were funded by Daiichi Sankyo, the manuscript of edoxaban, which may also cause potential source of bias [25]. And, this meta-analysis tested heterogeneity with the Cochrane Chi-square test and I^2^ statistics.

### Conclusion

A pooled meta-analysis of 5 prospective RCTs and a total of 31262 patients indicated that edoxaban seems to have a favorable safety profiles with respect to bleeding risk and mortality, in comparison to warfarin. However, further prospective RCTs are urgently needed to confirm the results of this meta-analysis.

## Supporting Information

Figure S1 **Forest Plot of risk ratios of bleeding events for comparison edoxaban with warfarin.** A series of forest plots of risk ratios (RRs) of bleeding events for comparison of given edoxaban or warfarin according to every trial were pooled. All 5 trials (n = 31152) reported events of major bleeding, clinically relevant nonmajor bleeding or minor bleeding and any bleeding, as well as 2 trials (n = 29256) reported events of fatal bleeding. CI confidence interval.
(TIF)

Figure S2 **Forest Plot of risk ratios of bleeding events for comparison edoxaban with warfarin.** A series of forest plots of risk ratios (RRs) of bleeding events for comparison of given edoxaban or warfarin according to every trial were pooled. Other than ENGAGE AF-TIMI 48, other 4 trials (n = 10,157) reported events of major bleeding, clinically relevant nonmajor bleeding, minor bleeding and any bleeding. CI confidence interval.
(TIF)

Figure S3 **Forest plots of studies for mortality for comparison edoxaban with warfarin.** Forest plots of studies for mortality of all causes or cardiovascular disease for comparison edoxaban with warfarin. Other than ENGAGE AF-TIMI 48, two trials (n = 9,386) reported available data. CVD denotes cardiovascular disease. CI confidence interval.
(TIF)

Table S1 **Search criterion of Medline (via PubMed, from inception to March 8, 2014).**
(DOCX)

Table S2 **Search criterion of Embase (via OVID, from 1966 to 2014).**
(DOCX)

Table S3 **Search criterion of Web of Science (from 1984 to 2014).**
(DOCX)

Table S4 **Characteristics of excluded full-text studies.**
(DOCX)

Checklist S1 **PRISMA 2009 Checklist.**
(DOC)
