# Supplementary figures and images for: Bleeding Risk and Mortality of Edoxaban: A Pooled Meta-Analysis of Randomized Controlled Trials
Source: PLoS One. 2014 Apr 15;9(4):e95354. doi: 10.1371/journal.pone.0095354 (PMC3988190; doi:10.1371/journal.pone.0095354)

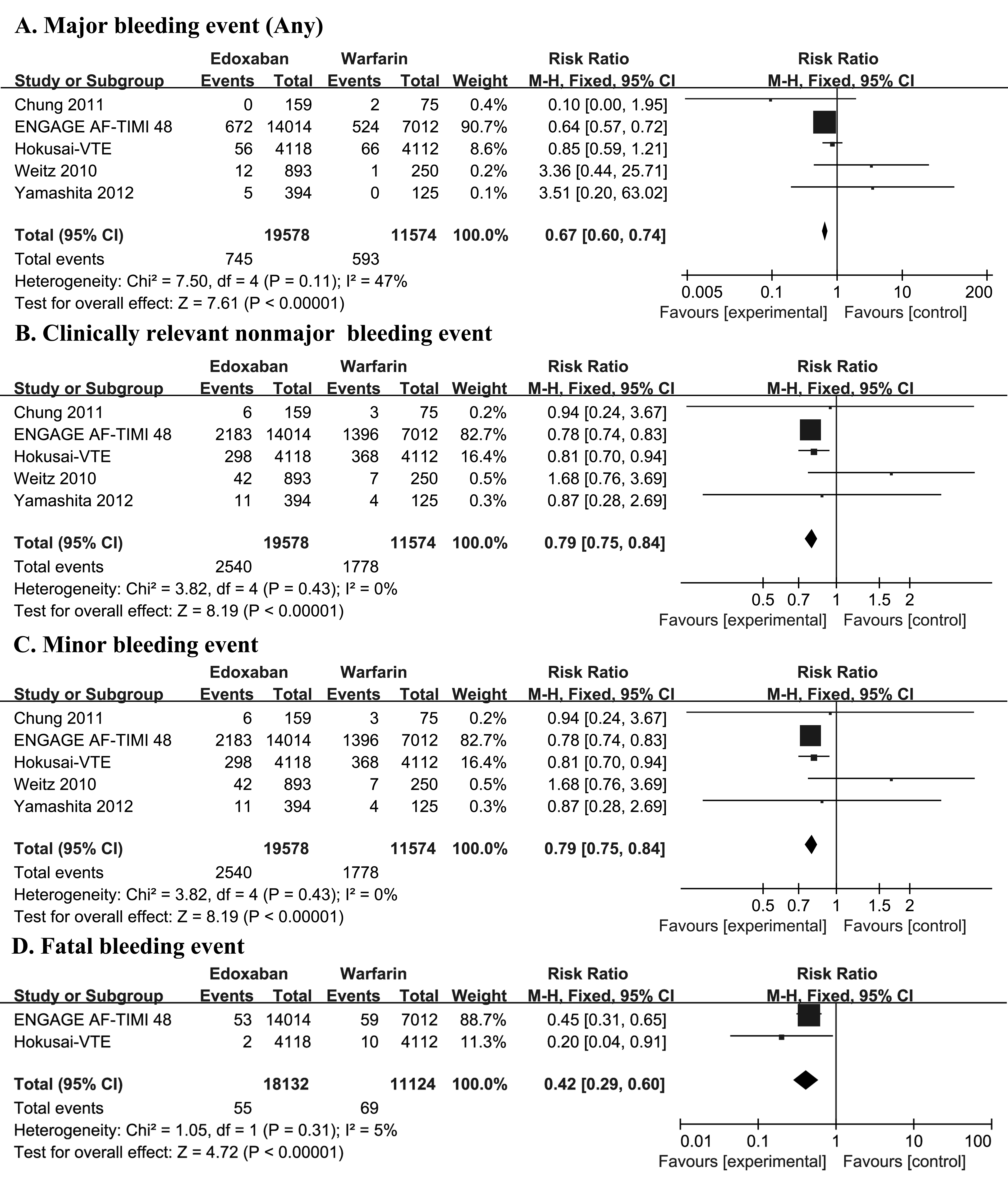

Supplement: Figure S1 — Forest Plot of risk ratios of bleeding events for comparison edoxaban with warfarin. A series of forest plots of risk ratios (RRs) of bleeding events for comparison of given edoxaban or warfarin according to every trial were pooled. All 5 trials (n = 31152) reported events of major bleeding, clinically relevant nonmajor bleeding or minor bleeding and any bleeding, as well as 2 trials (n = 29256) reported events of fatal bleeding. CI confidence interval. (TIF) [file pone.0095354.s001.tif]

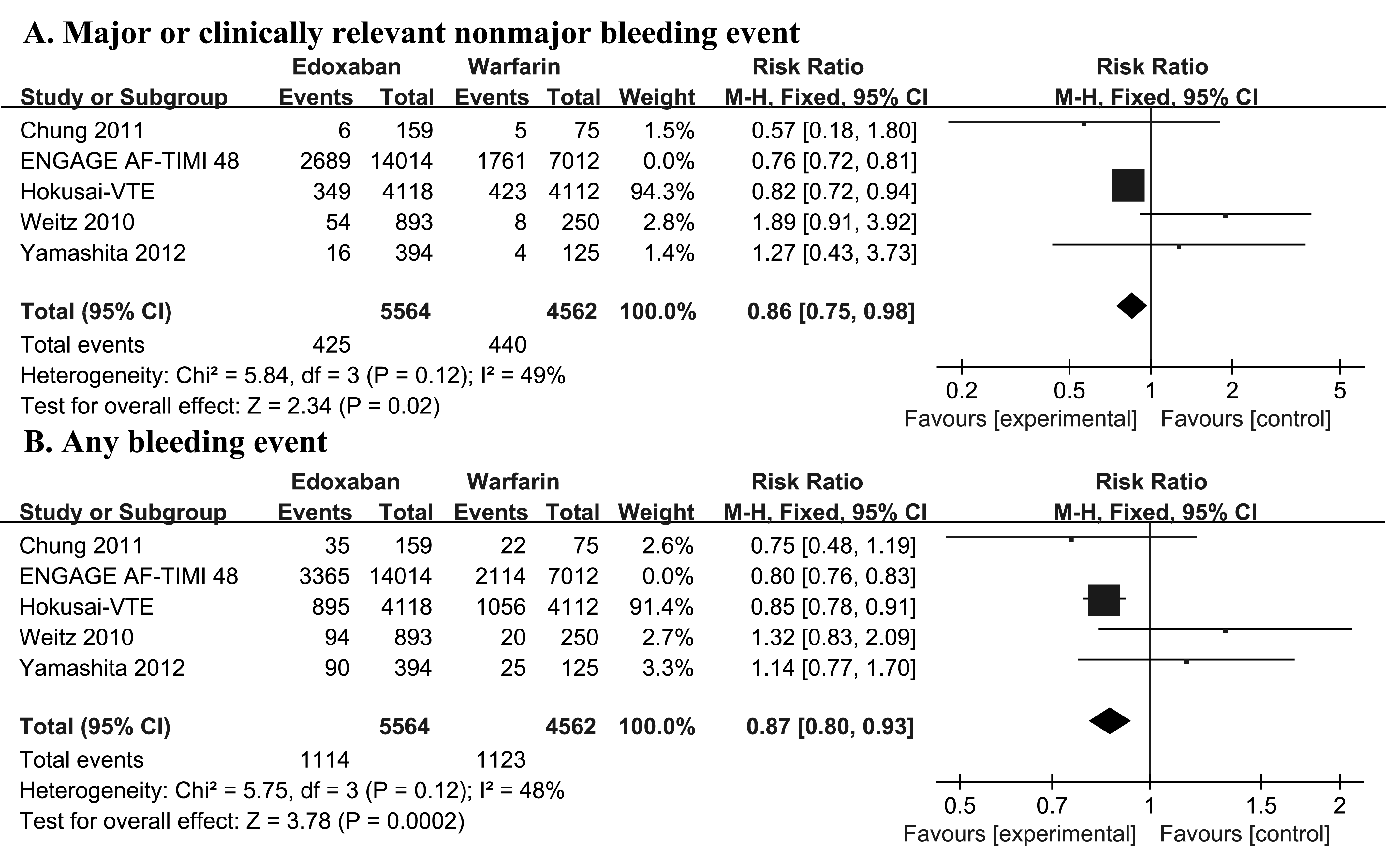

Supplement: Figure S2 — Forest Plot of risk ratios of bleeding events for comparison edoxaban with warfarin. A series of forest plots of risk ratios (RRs) of bleeding events for comparison of given edoxaban or warfarin according to every trial were pooled. Other than ENGAGE AF-TIMI 48, other 4 trials (n = 10,157) reported events of major bleeding, clinically relevant nonmajor bleeding, minor bleeding and any bleeding. CI confidence interval. (TIF) [file pone.0095354.s002.tif]

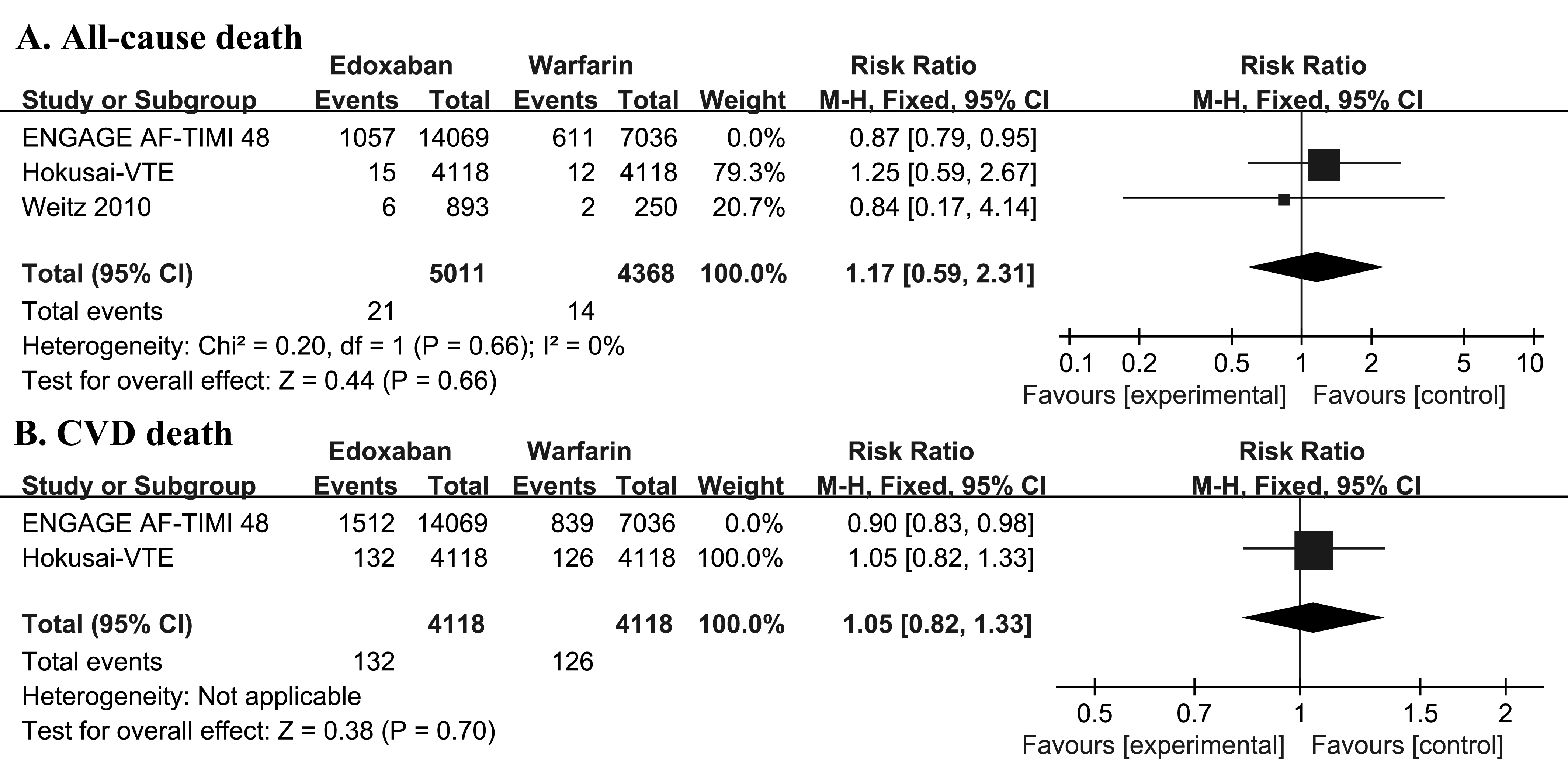

Supplement: Figure S3 — Forest plots of studies for mortality for comparison edoxaban with warfarin. Forest plots of studies for mortality of all causes or cardiovascular disease for comparison edoxaban with warfarin. Other than ENGAGE AF-TIMI 48, two trials (n = 9,386) reported available data. CVD denotes cardiovascular disease. CI confidence interval. (TIF) [file pone.0095354.s003.tif]
