# Supplementary material for: Bleeding Risk and Mortality of Edoxaban: A Pooled Meta-Analysis of Randomized Controlled Trials
Source: PLoS One. 2014 Apr 15;9(4):e95354. doi: 10.1371/journal.pone.0095354 (PMC3988190; doi:10.1371/journal.pone.0095354)
Supplement: Table S1 — Search criterion of Medline (via PubMed, from inception to March 8, 2014). (DOCX) [file pone.0095354.s004.docx]

Table S1 Search criterion of Medline (via Pubmed, from inception to March 8, 2014)

| No. | Query Results | Results |
| --- | --- | --- |
| #20 | Search ((((((((((new oral anticoagulants[Title/Abstract]) OR factor Xa inhibitors[Title/Abstract]) OR edoxaban[Title/Abstract])) OR Edoxaban[MeSH]) OR #6) OR edoxaban[MeSH Major Topic])) AND (((((warfarin[Title/Abstract]) OR coumadine[Title/Abstract]) OR coumadin[Title/Abstract]) OR marevan[Title/Abstract]) OR warfarin[MeSH Major Topic]))) AND ((random*[Title/Abstract]) OR ("Randomized Controlled Trials as Topic"[Mesh] OR "Randomized Controlled Trial" [Publication Type])) | 98 |
| #19 | Search (random*[Title/Abstract]) OR ("Randomized Controlled Trials as Topic"[Mesh] OR "Randomized Controlled Trial" [Publication Type]) | 839728 |
| #18 | Search random*[Title/Abstract] | 701069 |
| #17 | Search "Randomized Controlled Trials as Topic"[Mesh] OR "Randomized Controlled Trial" [Publication Type] | 444429 |
| #15 | Search ((((((((new oral anticoagulants[Title/Abstract]) OR factor Xa inhibitors[Title/Abstract]) OR edoxaban[Title/Abstract])) OR Edoxaban[MeSH]) OR #6) OR edoxaban[MeSH Major Topic])) AND (((((warfarin[Title/Abstract]) OR coumadine[Title/Abstract]) OR coumadin[Title/Abstract]) OR marevan[Title/Abstract]) OR warfarin[MeSH Major Topic]) | 412 |
| #14 | Search ((((warfarin[Title/Abstract]) OR coumadine[Title/Abstract]) OR coumadin[Title/Abstract]) OR marevan[Title/Abstract]) OR warfarin[MeSH Major Topic] | 17841 |
| #13 | Search warfarin[MeSH Major Topic] | 8190 |
| #12 | Search marevan[Title/Abstract] | 12 |
| #11 | Search coumadin[Title/Abstract] | 901 |
| #10 | Search coumadine[Title/Abstract] | 15 |
| #9 | Search warfarin[Title/Abstract] | 15782 |
| #8 | Search ((((((new oral anticoagulants[Title/Abstract]) OR factor Xa inhibitors[Title/Abstract]) OR edoxaban[Title/Abstract])) OR Edoxaban[MeSH]) OR #6) OR edoxaban[MeSH Major Topic] | 1270 |
| #7 | Search edoxaban[MeSH Major Topic] | 0 |
| #6 | Search Edoxaban[MeSH] Schema: all | 0 |
| #5 | Search Edoxaban[MeSH] | 0 |
| #4 | Search ((new oral anticoagulants[Title/Abstract]) OR factor Xa inhibitors[Title/Abstract]) OR edoxaban[Title/Abstract] | 1270 |
| #3 | Search edoxaban[Title/Abstract] | 161 |
| #2 | Search factor Xa inhibitors[Title/Abstract] | 688 |
| #1 | Search new oral anticoagulants[Title/Abstract] | 595 |
