# Supplementary material for: Bleeding Risk and Mortality of Edoxaban: A Pooled Meta-Analysis of Randomized Controlled Trials
Source: PLoS One. 2014 Apr 15;9(4):e95354. doi: 10.1371/journal.pone.0095354 (PMC3988190; doi:10.1371/journal.pone.0095354)
Supplement: Table S2 — Search criterion of Embase (via OVID, from 1966 to 2014). (DOCX) [file pone.0095354.s005.docx]

Table S2 Search criterion of Embase (via OVID, from 1966 to 2014)

| No. | Query Results | Results |
| --- | --- | --- |
| #17 | #5 AND #11 | 601 |
| #16 | #5 AND #11 AND #15 | 180 |
| #15 | #12 OR #13 OR #14 | 964281 |
| #14 | random*:ab,ti | 860105 |
| #13 | 'randomized controlled trial (topic)'/exp | 46844 |
| #12 | 'randomized controlled trial'/exp | 336353 |
| #11 | #6 OR #7 OR #8 OR #9 OR #10 | 29955 |
| #10 | 'warfarin'/exp/mj | 17536 |
| #9 | marevan:ab,ti | 16 |
| #8 | coumadin:ab,ti | 1443 |
| #7 | coumadine:ab,ti | 49 |
| #6 | warfarin:ab,ti | 22358 |
| #5 | #1 OR #2 OR #3 OR #4 | 1874 |
| #4 | edoxaban/exp/mj | 162 |
| #3 | 'factor xa inhibitors':ab,ti | 863 |
| #2 | 'new oral anticoagulants':ab,ti | 948 |
| #1 | edoxaban:ab,ti | 267 |
