# Supplementary material for: Bleeding Risk and Mortality of Edoxaban: A Pooled Meta-Analysis of Randomized Controlled Trials
Source: PLoS One. 2014 Apr 15;9(4):e95354. doi: 10.1371/journal.pone.0095354 (PMC3988190; doi:10.1371/journal.pone.0095354)
Supplement: Table S3 — Search criterion of Web of Science (from 1984 to 2014). (DOCX) [file pone.0095354.s006.docx]

Table S3 Search criterion of Web of Science (from 1984 to 2014)

| No. | Query Results | Results |
| --- | --- | --- |
| #5 | #3 AND #2 AND #1 Indexes=SCI-EXPANDED, SSCI, A&HCI, CPCI-S, CCR-EXPANDED, IC Timespan=1986-2014 | 409 |
| #4 | #2 AND #1 Indexes=SCI-EXPANDED, SSCI, A&HCI, CPCI-S, CCR-EXPANDED, IC Timespan=1986-2014 | 1062 |
| #3 | TOPIC: (random*) OR TOPIC: (randomized controlled trial) OR TOPIC: (randomized controlled trials) Indexes=SCI-EXPANDED, SSCI, A&HCI, CPCI-S, CCR-EXPANDED, IC Timespan=1986-2014 | 997537 |
| #2 | TOPIC: (warfarin) OR TOPIC: (marevan) OR TOPIC: (coumadin) OR TOPIC: (coumadine) Indexes=SCI-EXPANDED, SSCI, A&HCI, CPCI-S, CCR-EXPANDED, IC Timespan=1986-2014 | 16882 |
| #1 | TOPIC: (edoxaban) OR TOPIC: (factor Xa inhibitors) OR TOPIC: (new oral anticoagulants) Indexes=SCI-EXPANDED, SSCI, A&HCI, CPCI-S, CCR-EXPANDED, IC Timespan=1986-2014 | 5409 |
