# Supplementary material for: Bleeding Risk and Mortality of Edoxaban: A Pooled Meta-Analysis of Randomized Controlled Trials
Source: PLoS One. 2014 Apr 15;9(4):e95354. doi: 10.1371/journal.pone.0095354 (PMC3988190; doi:10.1371/journal.pone.0095354)
Supplement: Table S4 — Characteristics of excluded full-text studies. (DOCX) [file pone.0095354.s007.docx]

Table S4 Characteristics of excluded full-text studies

| **Study*** | **Reason for exclusion** |
| --- | --- |
| Mendell J, 2013 [[1](#_ENREF_1)] | phase 1 study, healthy subjects, too small (n=72), short duration‡: 5 days  no available data of outcomes that defined in this meta-analysis† |
| Raskob G, 2013 [[2](#_ENREF_2)] | Secondary analysis of Hokusai-VTE that included in our study |
| Ogata K, 2010 [[3](#_ENREF_3)] | phase 1 study, healthy subjects, no available data of outcomes that defined in this meta-analysis† |
| Fuji T, 2010 [[4](#_ENREF_4)] | short duration‡: 11–14 days. |
| STARS E-3, 2010 [[5](#_ENREF_5)] | the control drug is enoxaparin sodium, short duration‡: 2 weeks |
| STARS J-4, 2010 [[6](#_ENREF_6)] | the control drug is enoxaparin sodium, short duration‡: 2 weeks |
| Raskob G, 2010 [[7](#_ENREF_7)] | the control drug is dalteparin, short duration‡: 7–10 days |
| Koretsune Y, 2009 [[8](#_ENREF_8)] | pharmacokinetics and pharmacodynamics study, no available data of outcomes that defined in this meta-analysis† |
| Yasaka M, 2009 [[9](#_ENREF_9)] | The endpoint is intracranial hemorrhage, no available data of outcomes that defined in this meta-analysis† |
| Zafar MU, 2007 [[10](#_ENREF_10)] | phase 1 study, healthy subjects, too small (n=12), no available data of outcomes that defined in this meta-analysis† |

* Study was named as first author plus publication year or the registered name of clinical trial

† The safety end-points of our meta-analysis was bleeding events (any overt bleeding, major or clinically relevant non-major bleeding event, minor bleeding or any bleeding events) or mortality (all-cause death, cardiovascular death) for patients received edoxaban or warfarin.

‡ Duration in our study is ≥ 3 months

**References:**

1. Mendell J, Noveck RJ, Shi M (2013) A randomized trial of the safety, pharmacokinetics and pharmacodynamics of edoxaban, an oral factor Xa inhibitor, following a switch from warfarin. Br J Clin Pharmacol 75: 966-978.

2. Raskob G.E. BH, Angchaisuksiri P., Oh D., Boda Z., Lyons R.M., Weitz J.I., Zhang G., Lanz H.J., Mercuri M. (2013) Edoxaban For Long-Term Treatment Of Venous Thromboembolism In Cancer Patients. Blood 122: 211.

3. Ogata K, Mendell-Harary J, Tachibana M, Masumoto H, Oguma T, et al. (2010) Clinical Safety, Tolerability, Pharmacokinetics, and Pharmacodynamics of the Novel Factor Xa Inhibitor Edoxaban in Healthy Volunteers. Journal of Clinical Pharmacology 50: 743-753.

4. Fuji T, Fujita S, Tachibana S, Kawai Y (2010) A dose-ranging study evaluating the oral factor Xa inhibitor edoxaban for the prevention of venous thromboembolism in patients undergoing total knee arthroplasty. J Thromb Haemost 8: 2458-2468.

5. Fuji T WC-J, Fujita S (2010) Edoxaban versus enoxaparin for thromboprophylaxis after total knee arthroplasty: the STARS E-3 trial. Pathophysiol Haemost Thromb 37 Suppl: OC297.

6. Fujita S FT, Tachibana S (2010) Safety and efficacy of edoxaban in patients undergoing hip fracture surgery Pathophysiol Haemost Thromb 37 Suppl 37 Suppl.: P366.

7. Raskob G, Cohen AT, Eriksson BI, Puskas D, Shi MG, et al. (2010) Oral direct factor Xa inhibition with edoxaban for thromboprophylaxis after elective total hip replacement. Thrombosis and Haemostasis 104: 642-649.

8. Koretsune Y, Inoue H, Kawai Y, Uchiyama S, Yamaguchi T (2009) The Oral Factor Xa Inhibitor DU-176b in Japanese Warfarin-Naive Patients With Atrial Fibrillation: Results of Two Phase II Open-Label, Dose-Escalation Studies. Journal of the American College of Cardiology 53: A430-A431.

9. Yasaka M, Inoue H, Kawai Y, Yamaguchi T, Uchiyama S, et al. (2009) Asymptomatic Intracranial Hemorrhage Among Japanese Patients Taking Edoxaban for Non-Valvular Atrial Fibrillation. Blood 114: 441-441.

10. Zafar MU, Vorchheimer DA, Gaztanaga J, Velez M, Yadegar D, et al. (2007) Antithrombotic effects of factor Xa inhibition with DU-176b: Phase-I study of an oral, direct factor Xa inhibitor using an ex-vivo flow chamber. Thrombosis and Haemostasis 98: 883-888.
